# Using quality improvement to close HIV prevention gaps and strengthen district health systems: Blantyre, Malawi's approach and early implementation

**DOI:** 10.3389/frph.2025.1558630

**Published:** 2025-06-19

**Authors:** Sara M. Allinder, Edward Moses, Moses Enock, Gift Kawalazira, Rose Nyirenda, Andrew Gonani, Yohane Kamgwira, Bruce Agins, Richard Birchard, Joseph Murungu, Deborah Hoege, Charles B. Holmes, Martias Joshua

**Affiliations:** ^1^ Center for Innovation in Global Health, Georgetown University, Washington, DC, United States; ^2^ Independent Consultant, Lilongwe, Malawi; ^3^ Blantyre District Health Office, Blantyre, Malawi; ^4^ Directorate of HIV/AIDS, STIs, and Viral Hepatitis, Malawi Ministry of Health, Lilongwe, Malawi; ^5^ Quality Management Directorate, Ministry of Health, Lilongwe, Malawi; ^6^ Malawi National AIDS Commission, Blantyre, Malawi; ^7^ HEALTHQUAL, UCSF Institute for Global Health Sciences, New York, NY, United States; ^8^ HEALTHQUAL, UCSF Institute for Global Health Sciences, Harare, Zimbabwe; ^9^ Health Services (Reforms), Ministry of Health, Lilongwe, Malawi

**Keywords:** HIV, prevention, HIV prevention, quality improvement (QI), health system, sub-national, district-based model, Malawi

## Abstract

Application of quality improvement (QI) methodology to HIV prevention is relatively nascent but has the potential to transform national and local programs. In Blantyre, Malawi, a unique government-led partnership known as the Blantyre Prevention Strategy (BPS) is applying QI as a core element of a cohesive sub-national HIV prevention system. BPS launched a QI collaborative (QIC) in early 2021—the first of its kind dedicated to HIV prevention within a health system context—focused on scale-up of pre-exposure prophylaxis (PrEP) to prevent HIV. Known as PrEPUp!, the QIC included 23 participating facilities—representing the public and private sectors, drop-in centers for key populations, and a tertiary education clinic—and has become the major platform for facility teams to exchange knowledge and share progress. Based on the implementation of QI activities, interventions identified for testing resulted in health center system modifications that promotes increased uptake of PrEP. In addition, knowledge generated through the QIC informs other stakeholders, including implementing partners funded by the U.S. President's Emergency Plan for AIDS Relief (PEPFAR), and improves coordination and mentoring executed by the Blantyre District Health Office (DHO). In a departure from traditional QI, BPS has engaged community labs that generate insights from clients and other influential stakeholders about demand and service access barriers and has connected those labs with facilities through learning sessions. This approach has been widely lauded locally in Malawi and is being adapted in Lilongwe District to underpin implementation science for injectable PrEP.

## Introduction

Blantyre District's HIV prevalence rate measured 17.7% in 2015/2016—twice the national rate—and there were high rates of modeled incidence (1). A rapidly urbanizing and growing district of more than 1.5 million people, barriers to effective HIV care and prevention in Blantyre included fragmented delivery and lack of visibility of partner activities, misaligned and short-term program focus, weak data systems and insufficient data use, poor risk assessments and targeting, and inconsistent community demand (2). Primary prevention interventions, from condoms to oral pre-exposure prophylaxis (PrEP) to multi-sectoral interventions for adolescent girls and young women (AGYW), had not yet met their public health potential in Malawi. In 2020, the Government of Malawi collaborated with global and local experts to launch the Blantyre Prevention Strategy (BPS), aimed at strengthening a locally led public health management system in Blantyre capable of effective, efficient, and high-quality HIV prevention service delivery, including novel products such as PrEP, an intervention proven to prevent nearly all HIV infections when used correctly.

Integral to the strategy was co-development of a quality improvement (QI) approach to HIV prevention led by the district in coordination with central authorities. Globally, QI methods have been widely applied in the health care sector, including for HIV care and treatment, but they had not been routinely applied to HIV prevention prior to 2021 (3, 4). However, as incidence rates stall at unacceptably high rates and progress toward Sustainable Development Goal targets languish, new approaches are needed to address persistent challenges in the uptake and effectiveness of HIV prevention programs (5).

QI involves a systems approach to continuously improve patient experiences and outcomes. The Healthcare Quality Improvement Partnership describes the three dimensions of quality as clinical effectiveness—care delivered following the best evidence to improve an individual's health outcomes; patient experience—care that gives patients as positive an experience in receiving care as possible by honoring patients' wants and needs with compassion, dignity, and respect; and patient safety—providing care while avoiding harm and risk to the individual's safety (6). QI collaboratives (QICs) gather groups of professionals from within organizations or externally to jointly improve health services. Collaboratives use a structured approach involving a standardized set of measures focusing on a specific aim and deploying rapid cycles of change to achieve these improvements (6).

Health care QI is high on the global health agenda. The 2018 *Lancet Global Health Commission on High Quality Health Systems in the SDG Era* called for health systems that can measure and use data to learn, provide timely and effective health promotion and prevention services, and prevent and detect disease early (7). As part of a future research agenda, the commission identified the need to design and test district-level learning strategies, such as QI collaboratives, management innovations, and “intrinsic and extrinsic approaches to motivate providers.” (7) Similarly, key operational levers cited in the World Health Organization's (WHO) Primary Health Care Monitoring Framework include “systems at the local, subnational and national levels to continuously assess and improve the quality of integrated health services” and “models of care that promote high-quality, people-centered primary care and essential public health functions as the core of integrated health services throughout the course of life” (8).

This paper provides a case study in how QI methodology, specifically a QIC approach, was applied in Blantyre as an innovative technique to support sub-national leadership in strengthening HIV prevention. It will outline how BPS adapted an approach that was largely untested in HIV prevention, created the structures and processes needed for quality PrEP delivery in Blantyre, and provided the inputs to facilitate the strengthening of the sub-national system. It also explores the project's outputs, processes, and early experiences with implementation, including the broader impacts of the QI approach on district health leadership, national health objectives, and the sustainable integration of QI-driven HIV prevention services into district systems. The paper illustrates how the QI approach offers an innovative method to increase data access and use, utilize community and client insights to improve service delivery and demand generation, and address facility and provider barriers to PrEP uptake and continuation.

### Context

Malawi has been at the forefront of addressing the HIV epidemic, approaching the UNAIDS 95-95-95 targets with commendable progress (9). Yet, in 2020, as the BPS QIC approach was being co-developed in Blantyre, the country reported approximately 21,000 new HIV infections, underscoring the ongoing challenge in curbing the epidemic (10). The main driver of Malawi's HIV epidemic was the southern region of the country, including Blantyre which had a prevalence rate of 14.2% (10). Recognizing the critical need for effective prevention strategies, the Malawian government adopted PrEP as a national strategy in April 2019 and updated its National Prevention Strategy in 2020 (11). The December 2020 dissemination of the national oral PrEP guidelines refocused the country's public health strategy to key populations (KPs) such as men who have sex with men (MSM), female sex workers (FSW), and transgender individuals, along with vulnerable groups like AGYW (12). To be successful, the new direction required public health systems to capable of putting these guidelines into practice.

Led by the Government of Malawi, with support from the Center for Innovation in Global Health at Georgetown University and other partners and funding from the Bill & Melinda Gates Foundation, BPS was designed to develop an effective and sustainable district-based approach to HIV prevention by strengthening core functional and management capabilities for deploying HIV prevention technologies within Blantyre's existing public health system. One of its core pillars was improving the district's HIV prevention service delivery by building QI capacity using a QIC approach.

The BPS strategy was centered on the Blantyre District Health Office (DHO) and, for the QI pillar, specifically building capacity and embedding systems in the QI unit to better coordinate resources and partners to align with Malawi's 2017 National Quality Policy (13). According to Malawi's National Quality Management Strategy, the structure for quality management at district level has an appointed District Quality Management Coordinator (QI Coordinator) appointed who oversees all quality management initiatives in the district. Each public health facility has a designated Quality Management focal person who reports to the QI Coordinator.

The QI Coordinator is responsible for marshalling the diverse array of resources and partners essential to address quality of services in Blantyre's public health system—comprising 37 public health centers, as well as private service delivery and non-profit drop-in centers for KPs. At the initiation of BPS, in addition to the coordinator, the DHO had a deputy QI coordinator and 20 QI coaches. Like the QI Coordinator, these district-level coordinators are nurses or clinicians who also work in health care facilities and serve as a technical focal person for a designated public health area [e.g., sexually transmitted infections (STI)] as district health officers.

With the aim of increasing QI coaching dosage and reach, the PrEPUP! QIC encouraged changes to the district's existing QI structure. First, with the support of the Ministry of Health (MoH) Quality Management Directorate's (QMD) zonal offices, BPS recognized QI mentors by adding QI coaching to each district health coordinator's job responsibilities, expanding the number of staff trained to provide QIC coaching and supporting sustainability from the outset. Building the 20 QI coaches' capacity enabled expansion of QI coaching to additional health facilities, which could not be achieved by the district coordinator alone. Secondly, the PrEPUp! QIC took advantage of existing health facility clusters that were formed originally for data quality reviews and audits to maximize efficiency. For each of the five clusters, four QI coaches oversee their QI project implementation with one serving as QI cluster lead. All facilities in a cluster report their QI projects to the QI cluster lead who in turn reports to the district QI Coordinator. Thirdly, changes were made to the District Quality Improvement Support Team (QIST) meetings to increase frequency from quarterly to monthly and include the QI coaches in addition to heads of departments and the QI Coordinator.

In addition, BPS utilized existing QI structures at the facility level. Each department in the facility has a Work Improvement Team (WIT), which reports to the facility QIST. The PrEPUP! QIC trained 81 facility leads in QI so that they can effectively lead and facilitate the WITs' and QISTs' QI initiatives.

### Implementation

#### Development of the BPS quality improvement collaborative methodology

BPS leveraged the expertise of UCSF-HEALTHQUAL to support the application and co-development of QI methodology based on its experience building capacity of national quality programs in Malawi and other countries. The QI component of BPS was based on the UCSF-HEALTHQUAL framework for capacity-building (14) and the Breakthrough Series Collaborative model (15), which is designed to rapidly improve a specific aspect of service delivery. Although the QI collaborative methodology was not new to Malawi, its integration into routine activities of the district was innovative and aligned with the strong desire of the Blantyre district leadership to promote district-wide knowledge exchange and sharing (16).

The design of the BPS QIC was adapted for PrEP adhering to five essential elements: (1) PrEP was cited as the specified intervention for which measures were developed, (2) clinical experts and experts in QI were identified to provide ideas and coaching support for improvement, (3) multi-professional teams from all sites authorized to deliver PrEP at the start of 2021 participated, (4) the “Model for Improvement” and its Plan-Do-Study-Act (PDSA) methodology was adopted (17–19); and (5) the collaborative process involved a series of structured activities, which included learning sessions. The DHO and partners also worked together to ensure coordination and connection between BPS's QI and human-centered design (HCD) components, ensuring that insights from one were integrated into the work of the other.

**Figure 1:**
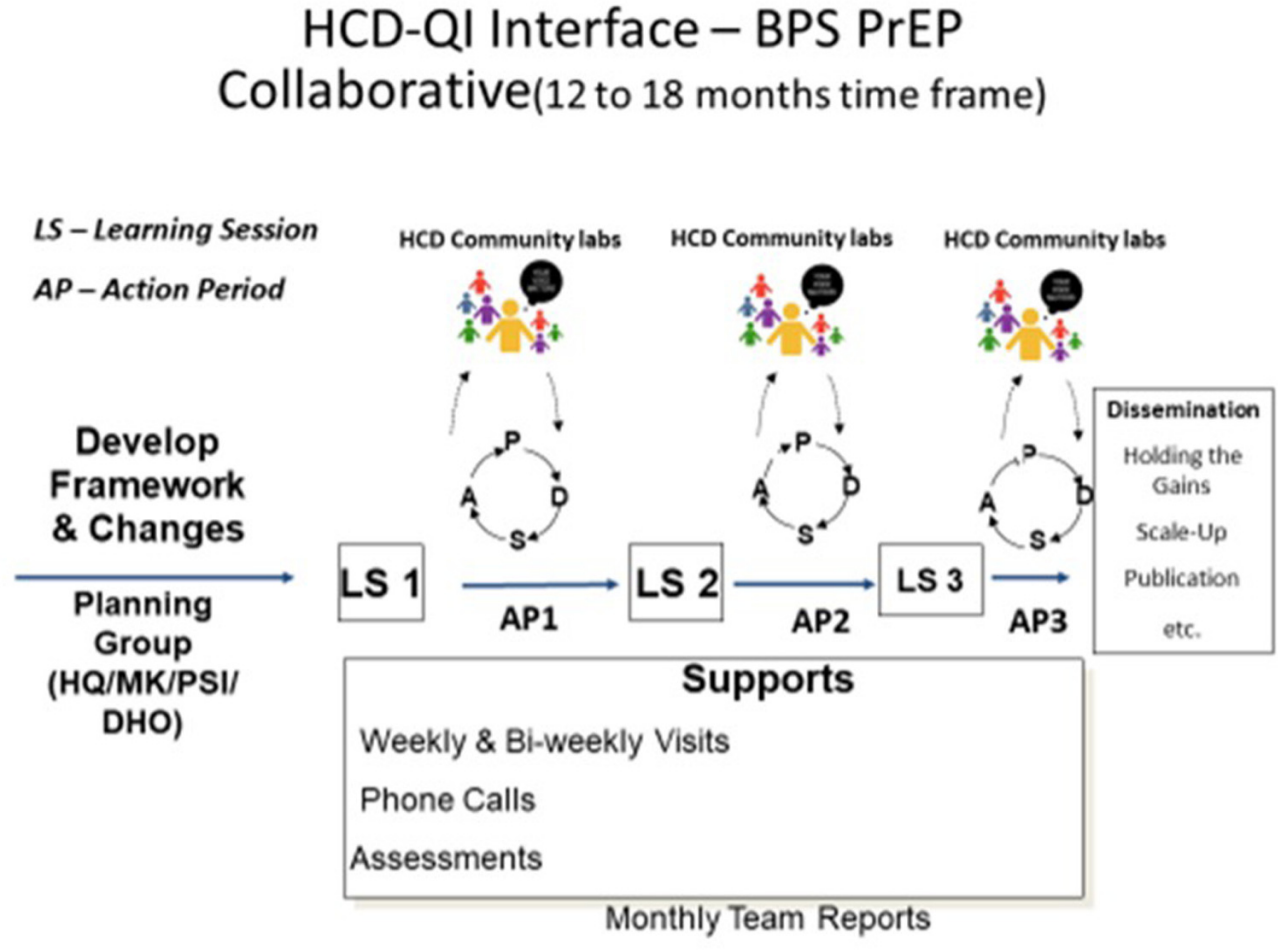
BPS quality improvement collaborative methodology (the planning group included UCSF-HEALTHQUAL, MaiKhanda trust, population services international, and the DHO, i.e., HQ/MK/PSI/DHO).

The BPS QIC model included a formative co-development phase during which ideas collected from local stakeholders were used in the initial planning meeting and to adapt the approach to local contexts. During this period, preparation activities were launched with an emphasis on pre-work undertaken by DHO coordinators and the QMD with mentorship from the local and international UCSF-HEALTHQUAL team (Figure 1). Five learning sessions were convened over the course of 2.5 years for knowledge exchange, punctuated by action periods in which site-level activities were conducted, including process mapping, root cause analysis, and testing of changes to inform how to adapt systems to effectively deliver PrEP services in health care facilities.

#### Baseline data collection

In partnership with QMD and the Blantyre DHO, UCSF-HEALTHQUAL conducted a baseline assessment to evaluate quality management structures and capacity as well as readiness to provide PrEP services and apply QI methods. A DHO-led team used two data collection tools, one which assessed the quality program at each site and one which assessed the readiness to deliver PrEP. The PrEP readiness assessment included items on availability of HIV prevention and HIV services, access to guidelines, and availability of PrEP-trained providers. The assessment team interviewed health facility staff (facility leadership, QI focal person, and members of QI team) and collated data to create a district-wide assessment report on facility readiness to offer PrEP and inform planning for national oral PrEP roll out.

A total of 19 facilities and 50 healthcare workers (HCWs) were included in the initial assessment. Key findings showed that HCWs in most facilities (38/50) required support using QI methods; only 11 of the 50 HCWs reported being skilled and competent in implementing QI activities in their facilities; and less than half (8/19) of facilities implementing QI initiatives had seen some improvement. All facilities (19) provided HIV and STI testing in early 2021; however, few (6/19) were providing PrEP. Fewer than half had a copy of the national PrEP guidelines (7/19), but most (15/19) facilities had at least one provider trained on the PrEP guidelines.

#### BPS quality improvement collaborative design

A multi-sectoral BPS Design Meeting, involving implementing partners, civil society, and community group participants, officially launched the QIC in April 2021. The gathering established a planning committee comprising national, city, and district-based staff from QMD; the MoH Department of HIV/AIDS, STI, and Viral Hepatitis (DHA); the DHO QI Coordinator; and staff from local technical and implementing partners.

The Blantyre DHO and Blantyre City Assembly originally selected 15 facilities to participate; an additional four facilities were added in April 2021 and four more were added by April 2023. Of those latter four, two lower-tier private sector facilities/clinics were included on the DHO recommendation to represent a wider array of facilities where people access PrEP in Blantyre. All told, the PrEPUp! collaborative included 23 sites including drop-in centers for FSW and MSM, private sites targeting high-risk clients, public sites, and a tertiary education clinic.

#### Mentorship & training

The baseline assessment informed QI coaching and training provided to facilities during the QIC; implementing partners and the MoH also used the results to aid their understanding of PrEP readiness at facility-level. All 20 QI coaches were trained to build coaching skills and the methods of planning and leading QICs; 81 QI team members from the initial 21 PrEP Up! sites completed QI trainings, 42 health workers were oriented on PrEPUp! data management, and 330 HCWs participated in facility-based, mentor-led QI orientations. During action periods between learning sessions, DHO coordinators conducted QI coaching (with QI expert team mentorship) to guide teams in the processes of problem identification, root cause analysis, identifying change ideas, implementing PDSA cycles, and organizing their quality management programs to support these activities. As their skills increased, coaches conducted visits independently. Supplemental QI training was provided for staff at the 21 original facilities to augment QI coaching and enhance QI knowledge among site staff.

**Figure 2:**
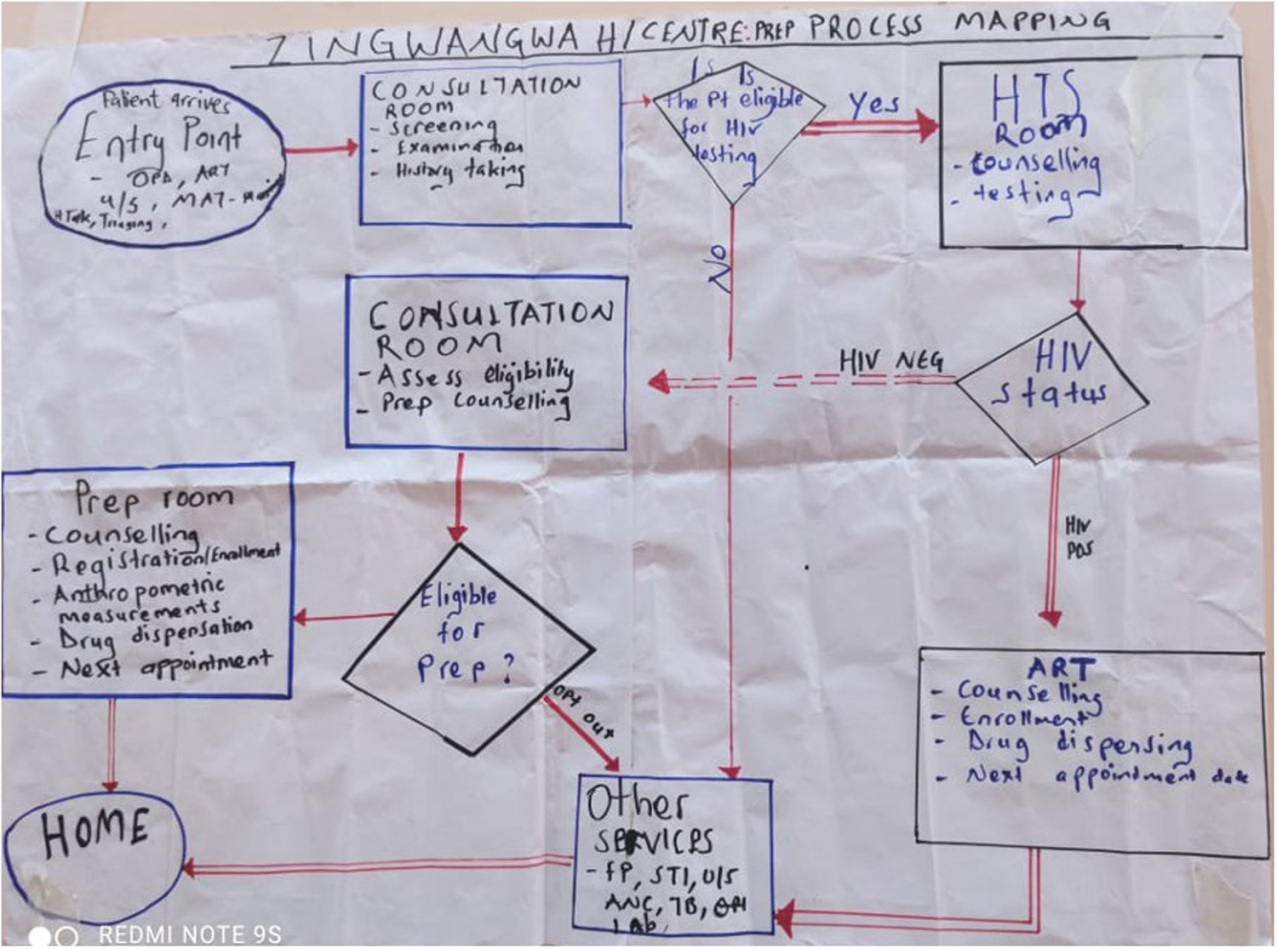
Zingwangwa health centre PrEP process map wall chart, photo taken September 27, 2021.

Performance data were collected monthly on the core set of PrEP QI indicators based on national guidelines and graphed on run charts. These indicators, which focused on PrEP introduction and scale up, complemented other epidemiologic and service delivery data collected through separate processes. Documentation of activities was captured on PDSA wall charts posted in each facility (Figure 2). Facility QI teams were guided to draw process maps to improve client flow, e.g., identifying people who would benefit from PrEP, eligibility assessment, HIV testing to exclude people living with HIV, and PrEP initiation. Improvement activities and data were shared at DHO-led learning sessions.

#### Learning sessions

Five QIC learning sessions held between mid-2021 and 2023 provided a platform for peer knowledge exchange with the aim of accelerating implementation of successful interventions and facilitating a sustained community of practice. Successful interventions were identified as best practices and disseminated across facilities through storyboard presentations. QIC learning sessions also enabled dissemination of the national PrEP guidelines and strategy, enabling a two-way communication process in which the implementation practices and challenges were fed back to the QMD and DHA to inform guideline adaptation.

The first PrEPUp! learning session (LS) was held in September 2021. Topics included preparation for data collection, review of the measures, process mapping and client flow discussions, and presentations from civil society organizations that focused on the care of KPs, particularly FSW and MSM. Their experience provided useful information to general health facility providers for serving these populations in their integrated care systems. Key needs/gaps that arose included prioritizing stigma reduction, segmenting demand creation according to KP grouping, and service integration at health facilities.

The second LS was held in two phases in January-February 2022. While LS 1 oriented participants to the QI process through facility maps highlighting PrEP entry points and integration of services, LS 2 served as a platform for facilities to report on their QI work and change ideas for PrEP. Facilities shared experiences regarding PrEP implementation from the first action period and initial learning from PrEP-focused QI activities. Topics included improvement of PrEP initiation and challenges related to continuation of PrEP past one month. By LS 3 in July 2022, facilities demonstrated significant levels of competence with the QI approach both in terms of presentations and data collection reflecting workflow improvements, facility-based demand creation, better targeting of STI clients, and increased PrEP uptake (20).

Planning and leadership of the QIC learning sessions was fully transitioned to DHO staff by the fifth and final learning session in October 2023. Staff both led and managed the planning and execution of the meetings, including the preparation of storyboards by participating facilities.

### Outputs

Based on the implementation of QI activities, interventions identified for testing resulted in health center system modifications that promoted increased uptake of PrEP (20, 21). Major categories of successful interventions for integrating PrEP into existing health care facility services included: (1) integration/expanding access to PrEP by offering counseling and delivery at other entry points [e.g., Family Planning (FP) and STI clinics] instead of just the ART clinic; (2) providing PrEP information at entry points across facilities; (3) routine demand creation activities in facility common areas including health talks, videos, posters, and pamphlets; (4) improving sexual history taking; and (5) addressing stigma among and generated by HCWs, which has been identified as a key barrier to accessing services.

As part of a complementary work stream, BPS employed an HCD-driven “community lab model” to collect and act on insights from community representatives related to barriers to service access and health-seeking behavior affecting demand for PrEP and other HIV prevention services. Insights generated from the community labs informed QI service delivery interventions in facilities and joint QI activities conducted by clinical/community partnerships through the learning sessions and facility staff meetings. For example, insights gathered from community labs that engaged FSW and AGYW informed the introduction of PrEP ambassadors. The ambassadors–a peer-based model to drive PrEP demand at facilities–supported increased uptake of oral PrEP and improved linkages between facilities and communities (22). Implementation progress reflecting the inputs from these labs were shared at QIC learning sessions and fed back to the communities to show how their insights were used to improve service delivery and program design. By linking the QIC and community lab work streams, BPS facilitated creation of a hybrid model that blends citizen voices and provider knowledge to improve delivery of prevention services through problem identification, solution development, and testing that is sustainable and transferrable.

## Discussion

By fostering platforms for sharing information, the BPS QIC enabled a synergy of expertise and resources that ensured each facility could scale-up PrEP service delivery following national PrEP guidelines while demonstrating that QI could facilitate HIV prevention and PrEP delivery in healthcare settings. This approach marked a significant shift from traditional top-down implementation models, building local capacity and ownership to continuously monitor performance and discuss results to foster responsive testing of change ideas. The district-based quality program has become pivotal in this regard, evolving into a sustainable approach for not only executing current HIV prevention strategies but also adapting to future challenges and innovations in the field. The success of BPS's QI approach has become foundational to Blantyre's HIV prevention system including utilization of the QI platform at facilities as a locus of data review, analysis, and decision making at that level.

Key components of the BPS QI approach included:

• Understanding of key demand barriers through solicitation of community input;
• Embedding QI and other supportive capabilities in the existing governance and programmatic structures within the DHO to achieve sustainability;
• QI approaches, such as process mapping, foster successful PrEP integration into existing services at general health care facilities by analyzing clinic flow through identification of key entry points where candidates currently seek care;
• Regular demand creation activities in healthcare facilities shaped by community input including information posted in public areas, strengthening skills and encouraging routine sexual history taking, development of PrEP-specific data collection tools to inform improvement work, and addressing stigma; and
• Continuous and intensive mentoring from QI experts that helped the DHO develop both individual staff capabilities and organizational capacity to leverage QI methods, including QICs, as a strategy to improve and scale-up health services.

Through BPS QI, the DHO gained specific competencies including knowledge and skills to implement and guide QI activities. Using standardized coaching, DHO staff guided facilities to sustain QI structures and strengthen their use of data to improve service delivery. Facility presentations of QI projects, and the knowledge reflected in related peer-led critiques during storyboard rounds, demonstrated the effectiveness of DHO coaching efforts. District QI coordinators and mentors also applied QI methodology to other public health areas, including management of a cholera outbreak and service delivery related to cyclone Freddy in 2023. The improvements led several large private hospitals to ask the DHO for direct QI technical support, including QI training for their officers.

Teams in each facility developed the change ideas to be applied in their delivery systems, adapting them to their specific context and involving internal stakeholders. Most changes occurred through modifications to internal processes and systems, although some aspects of data collection and follow up with clients who missed appointments required consideration of external policies and systems. For example, existing PrEP registers and other documentation systems did not include fields for the PrEPUp! indicators, which required creating improvised solutions and advocacy to modify registers to accommodate new fields. In addition, national guidelines initially did not include policies for contacting people who missed PrEP appointments; BPS data resulted in support for virtual contact once poor rates of continuity were noted.

Through QI activities, DHO staff and providers now understand the need for facility-based demand creation; PrEP-specific data management systems that also include documentation of contact following missed appointments; redesign of flow of both people and data based on a HIV status-neutral approach; and identification and engagement of specific facility departments that interact with people who would benefit from PrEP.

### Institutionalizing and sustaining the QI processes

While the PrEPUP! QIC structure formally ended with the fifth learning session, QI mentorship and communities of practice continue in Blantyre. Two affinity groups (health area-specific communities of practice) were established to strengthen knowledge exchange about integrating PrEP into STI and FP. These groups meet virtually and in-person to discuss challenges and solutions to service delivery implementation and client engagement and comprise staff members from participating facilities and QI coaches who are also the DHO coordinators in these health areas. Group text chats facilitate quick peer input on questions. These groups provide a forum for peer learning and exchange in smaller groups and for shorter time periods, facilitating more opportunities for collaboration and reducing time away from clinical services. They will serve as models for establishing similar groups of other types of service providers and are intended to become sustainable components of the Blantyre DHO health system.

To support institutionalization, QI coaches' meetings were formally structured as QIST meetings in BPS's third year after the Blantyre District DHSS declared that facility QI structures should be the platform through which all government-led and partner-led projects in the facilities are conducted and held accountable. Transitioning to QIST meetings will ensure sustainability and aligns with the BPS vision of institutionalizing the district's quality management program. An additional benefit of the QIST is that it includes all partners supporting QI in the district, which enables sharing of best practices and ensures coordination of activities across multiple implementing partners and programs.

External technical expertise was critical to build capacity in the district and to lead and support the entire scope of QI activities, including coaching and the collaborative. As capacity was built at district and facility levels, UCSF-HEALTHQUAL transitioned from leading implementation to providing specialized technical assistance directed toward district quality infrastructure with diminishing site-level involvement. The participation of Malawian QI experts enabled appropriate translation of QI language and concepts into local languages as needed and alignment and buy-in from the national quality program. This pivotal transition to local leadership and implementation increases the likelihood that QI and QICs will be sustained through any future changes in the healthcare delivery system. BPS's focus on capacity building and technical assistance aligns with global health priorities for local ownership and Malawi's own decentralization policy. Embedding these capabilities and knowledge in sub-national units enables input from communities, providers, and other stakeholders fostering adaptation of national policies and guidelines to their specific contexts.

In addition, the BPS QI approach is replicable in settings other than Blantyre. For example, the BPS QI approach was adapted to Lilongwe for the introduction of injectable PrEP through Malawi's “PathToScale” implementation science initiative. Blantyre DHO coordinators served as technical experts and peer mentors to their counterparts in Lilongwe and supported capacitation efforts there. Some adjustments were made to the approach to adapt it to the local health system and address other contextual factors in Lilongwe, but the core of the BPS PrEPUP! model remained intact. Lilongwe adaptation also offered important learning that subsequently informed adjustments in Blantyre.

### Impact

While causality cannot be attributed to BPS-supported interventions, BPS-supported facilities in Blantyre saw a more rapid uptake in oral PrEP compared to other facilities in Blantyre (21, 23). However, the most significant impact of the QIC approach is the tangible improvement in service delivery, sustained community engagement and responsibility for HIV prevention, and embedding BPS learnings in the national quality framework. By equipping HCWs with QI skills, promoting peer learning, and emphasizing local ownership of the QI program, the QICs have enhanced the quality and effectiveness of HIV prevention services, which translates into better health outcomes for communities. The learning from Blantyre has become an integral part of the national quality framework*;* PrEPUp! updates are routinely presented at the quarterly quality management technical working group in which all partners supporting the MoH in QI are represented.

### Challenges to implementation & sustainability

Several obstacles were encountered during implementation of the PrEPUp! QIC highlighting areas that require attention and adaptation in future HIV prevention initiatives. One prominent challenge was the limited availability of resources, e.g., HIV and STI test kits and medications, to support PrEP delivery, which hindered implementation of PrEP services in some facilities. Resource constraints were addressed by the DHO, which requested supply chain management changes from Ministry-level, and through WhatsApp groups and linked networks formed around 14 public health facilities, which facilitated the sharing of commodities between public, private, and community organization sites.

Further, HCWs required training and mentorship to become knowledgeable about PrEP and sexual history-taking as well as to develop proficiency in QI methodologies. To address these needs, HEALTHQUAL trained and mentored 20 QI coaches and 81 QI facility leads and provided health facility QI orientation using the QMD QI training curriculum. HEALTHQUAL also partnered with the Elizabeth Glazer Pediatric AIDS Foundation (EGPAF) country team to provide training on the national PrEP guidelines in all PrEPUP! sites. In addition, the need for continued capacity building and mentorship remains an ongoing consideration. As partner organizations transition to more advisory roles, the responsibility for maintaining up-to-date knowledge about the latest information related to HIV prevention and for continuing the QIC activities lies with local health care teams. In addition to the affinity groups noted above, the DHO hosted cluster learning sessions to exchange QI learning and information about integration of PrEP into various services. Additional efforts are needed to raise awareness, reduce stigma, and promote PrEP uptake among key and vulnerable populations.

### Limitations

BPS's QI approach, and the findings and outcomes of using QICs, may not be fully generalizable to all sub-national HIV prevention responses. The success of this approach is influenced by the specific local dynamics and commitment of Blantyre DHO leadership, the structure of its health system, its district and facility teams, and the QMD and other national stakeholders. Nimble data systems that allow collection of real-time data to target gaps and advance improvement may not be available elsewhere to permit essential monitoring and evaluation. In 2025, BPS began developing a toolkit to enable other countries to adapt the BPS model and its program elements, e.g., QI, to their local context. Adapting it to different organizational systems and structures within other district health units may require tailored strategies based on current QI functionality, which would also influence the cost of implementation. While co-development to fit local context takes time and requires building relationships of trust with a shared vision for the outcome, initial adaptation to Lilongwe has demonstrated that the approach can be adopted elsewhere with co-developed processes involving local stakeholders.

While early results are promising, the long-term impact of the QIC approach is yet to be fully assessed. In addition, scaling up utilization of QICs for PrEP to cover a broader geographic area presents logistical challenges. Expanding its reach will require careful planning and resource allocation. Challenges include ensuring data quality and completeness and availability and retention of qualified HCWs. Not all Malawian HCWs have been trained as certified PrEP providers, which will be critical to ensuring that PrEP is a standard component of primary healthcare systems. Further, achieving meaningful community engagement as part of routine program implementation needs to be assured as part of healthcare strategic planning.

## Conclusion

New approaches are needed to address the persistent challenges to preventing new HIV infections. QI methodology has been underutilized for HIV prevention but is particularly important as facility-based and community-level delivery channels expand across health care systems, particularly in countries with robust PrEP programs. BPS's application of QI methodology to HIV prevention and use of QI collaboratives to accelerate the scale-up of PrEP in the public health system represents an innovative approach to improving the effectiveness and sustainability of HIV prevention. PrEPUp! has demonstrated that building district-based QI capacity for implementation and leadership embedded in the DHO structure is a feasible and successful strategy to advance the scale up of HIV prevention services. By building local capacity and empowering district-level health systems, QICs can pave the way for the seamless integration of HIV prevention services into district public health and healthcare systems. The BPS collaborative model has demonstrated early success in improving PrEP uptake and service quality. It has empowered local health care teams, fostered district leadership, improved client-informed care, and aligned with national decentralization objectives. It offers a replicable model for improving prevention services through district-based implementation to tackle public health priorities.

## Data Availability

The original contributions presented in the study are included in the article/Supplementary Material, further inquiries can be directed to the corresponding author.

## References

[1] Ministry of Health. Malawi Population-Based HIV Impact Assessment (MPHIA) 2015–2016: Final Report. Malawi Ministry of Health. (2018). Available at: https://phia.icap.columbia.edu/malawi-final-report/ (Accessed November 05, 2024).

[2] Kawalazira G Kamgwira Y Allinder SM Mablekisi C Nyirenda R Hoege D A health systems approach to more effective subnational HIV prevention: development of Malawi’s Blantyre prevention strategy. BMJ Glob Health. (2025) 10(2):e016880. 10.1136/bmjgh-2024-016880 40010778 PMC11865780

[3] Ngongo Bahati P Kidega W Ogutu H Odada J Bender B Fast P Ensuring quality of services in HIV prevention research settings: findings from a multi-center quality improvement pilot in East Africa. AIDS Care. (2010) 22(1):119–25. 10.1080/09540120903012569 20390489

[4] Urwitz V Vuylsteke B Apers H Hales D Wentzlaff-Eggebert M Nöstlinger C. A multicountry European study on succeed: a general quality improvement tool in HIV prevention. Health Promot Int. (2020) 35(5):935–46. 10.1093/heapro/daz081 33099280

[5] Joint United Nations Programme on HIV/AIDS (UNAIDS). Take the Rights Path World AIDS Day 2024 Report. (2024). Available at: https://rightspath.unaids.org/?_gl=1%2a9y7kpw%2a_ga_T7FBEZEXNC%2aMTczNjIwNzY3MC4xLjAuMTczNjIwNzY3MC42MC4wLjA (Accessed November 12, 2024).

[6] Fereday S. A Guide to Quality Improvement Tools. London: Healthcare Quality Improvement Partnership (HQIP) (2020). Available at: https://www.hqip.org.uk/wp-content/uploads/2021/01/Final-Quality-Improvement-QI-Tools-09-12-20.pdf (Accessed November 18, 2024).

[7] Kruk ME Gage AD Arsenault C Jordan K Leslie HH Roder-DeWan S High-quality health systems in the sustainable development goals era: time for a revolution. Lancet Glob Health. 2018;6(11):e1196–2l52. 10.1016/S2214-109X(18)30386-3 30196093 PMC7734391

[8] World Health Organization. Primary health care measurement framework: monitoring health systems through a primary health care lens. (2022). Available at: https://www-who-int.libproxy.lib.unc.edu/publications/i/item/9789240044210 (Accessed November 21, 2024).

[9] UNAIDS. Malawi country factsheet - 2023 data. (2024). Available at: https://www.unaids.org/en/regionscountries/countries/malawi (Accessed November 21, 2024).

[10] Ministry of Health Malawi. Malawi population-based HIV impact assessment 2020–2021: MPHIA 2020–2021. Final report. Lilongwe, Malawi: Ministry of Health. (2022). Available at: https://phia.icap.columbia.edu/wp-content/uploads/2022/12/241122_Mphia_Foreword.pdf

[11] Malawi National AIDS Commission. National HIV Prevention Strategy 2015–2020. (2014). Available at: https://www.prepwatch.org/wp-content/uploads/2022/07/National-Strategic-Plan-for-HIV-and-AIDS-2020-25-Final.pdf (Accessed December 01, 2024).

[12] Ministry of Health Malawi. National Guidelines for the Provision of Oral Pre-Exposure Prophylaxis for Individuals at Substantial Risk of HIV in Malawi. (2020). Available at: https://dms.hiv.health.gov.mw/dataset/50056785-ba78-4ec0-a8f1-74d7da43ade6/resource/f5be8a13-c3a9-46e8-afb6-fef0a0912c2f/download/malawi-national-prep-guidelines.pdf&ved=2ahUKEwie2YKF–GKAxWZE1kFHbl7KBsQFnoECBYQAQ&usg=AOvVaw2NtEzchaJRGO7PGndwb5Dh (Accessed December 02, 2024).

[13] Ministry of Health Malawi. Quality Management Policy or the Health Sector. (2017). Available at: https://npc.mw/wp-content/uploads/2020/07/quality-management-policy.pdf (Accessed December 07, 2024).

[14] Bardfield J Agins B Akiyamab M Basenero A Luphalac P Kaindjee-Tjitukac F A quality improvement approach to capacity building in low- and middle-income countries. AIDS. (2015) 29(Suppl 2):S179–86. https://healthqual.ucsf.edu/sites/g/files/tkssra931/f/a-quality-improvement-approach-to-capacity.pdf 10.1097/QAD.0000000000000719 26102629

[15] Institute for Healthcare Improvement. The Breakthrough Series: IHI’s Collaborative Model for Achieving Breakthrough Improvement. IHI Innovation Series white paper. Boston: Institute for Healthcare Improvement. (2003). Available at: https://www.ihi.org/resources/white-papers/breakthrough-series-ihis-collaborative-model-achieving-breakthrough#downloads (Accessed December 12, 2024).

[16] Kawalazira G Enock M Jere MM Moses E Tambala J Agins B Improving rapid ART initiation in Blantyre, Malawi, through implementation of a quality improvement collaborative. International AIDS Society 2019 Science Meeting Abstract. (2019). Available at: https://programme.ias2019.org/Abstract/Abstract/3021

[17] Associates in Process Improvement. Model for Improvement. Available at: https://www.apiweb.org/ (Accessed December 12, 2024).

[18] Institute for Healthcare Improvement. How to Improve: Model for Improvement. Available at: https://www.ihi.org/resources/how-improve-model-improvement (Accessed December 14, 2024).

[19] Basenero A Neidel J Ikeda DJ Ashivudhi H Mpariwa S Kamangu JWN Integrating hypertension and HIV care in Namibia: a quality improvement collaborative approach. PLoS One. (2022) 17(8):e0272727. 10.1371/journal.pone.0272727 35951592 PMC9371294

[20] Likumbo S Enock M Moses E Tambala J Mablekisi C Kamgwira Y Building sustainable district-based quality improvement (QI) capacity for HIV prevention in public health facilities in blantyre, Malawi. 2024 International AIDS Conference Abstract (WEPEE590) (2024). Available at: https://www.iasociety.org/sites/default/files/AIDS2024/abstract-book/AIDS-2024_Abstracts.pdf

[21] Allinder S Kawalazira G Likumbo S Banda C Kamgwira Y Matanje B Enabling effective HIV prevention through capacitated district-based HIV prevention systems. 2024 International AIDS Conference Satellite (SAT 063) (2024). Available at: https://plus.iasociety.org/webcasts/enabling-effective-hiv-prevention-through-capacitated-district-based-hiv-prevention

[22] Allinder S Saidi F. Malawi Injectable PrEP path to scale study. PrEP Connection Webinar. (2023). Available at: https://www.youtube.com/watch?v=eUev33hZAV0; https://www.prepwatch.org/wp-content/uploads/2023/06/June-2023-PLN-.pdf

[23] Likumbo S Enock M Moses E Tambala J Mablekisi C Kamgwira Y Building sustainable district-based Quality Improvement (QI) capacity for HIV prevention in public health facilities in Blantyre, Malawi. AIDS2024 Abstract (WEPEE590).

